# Functional Production of a Soluble and Secreted Single-Chain Antibody by a Bacterial Secretion System

**DOI:** 10.1371/journal.pone.0097367

**Published:** 2014-05-13

**Authors:** Chiu-Min Cheng, Shey-Cherng Tzou, Ya-Han Zhuang, Chien-Chiao Huang, Chien-Han Kao, Kuang-Wen Liao, Ta-Chun Cheng, Chih-Hung Chuang, Yuan-Chin Hsieh, Ming-Hong Tai, Tian-Lu Cheng

**Affiliations:** 1 Department of Aquaculture, National Kaohsiung Marine University, Kaohsiung, Taiwan; 2 Department of Biological Science and Technology, National Chiao Tung University, Hsin-Chu, Taiwan; 3 Department of Biomedical Science and Environmental Biology, Kaohsiung Medical University, Kaohsiung, Taiwan; 4 Graduate Institute of Medicine, Kaohsiung Medical University, Kaohsiung, Taiwan; 5 Institute of Biomedical Sciences, National Sun Yat-Sen University, Kaohsiung, Taiwan; 6 Cancer Center, Kaohsiung Medical University Hospital, Kaohsiung, Taiwan; Baker IDI Heart and Diabetes Institute, Australia

## Abstract

Single-chain variable fragments (scFvs) serve as an alternative to full-length monoclonal antibodies used in research and therapeutic and diagnostic applications. However, when recombinant scFvs are overexpressed in bacteria, they often form inclusion bodies and exhibit loss of function. To overcome this problem, we developed an scFv secretion system in which scFv was fused with osmotically inducible protein Y (osmY), a bacterial secretory carrier protein, for efficient protein secretion. Anti-EGFR scFv (αEGFR) was fused with osmY (N- and C-termini) and periplasmic leader sequence (pelB) to generate αEGFR-osmY, osmY-αEGFR, and pelB-αEGFR (control), respectively. In comparison with the control, both the osmY-fused αEGFR scFvs were soluble and secreted into the LB medium. Furthermore, the yield of soluble αEGFR-osmY was 20-fold higher, and the amount of secreted protein was 250-fold higher than that of osmY-αEGFR. In addition, the antigen-binding activity of both the osmY-fused αEGFRs was 2-fold higher than that of the refolded pelB-αEGFR from inclusion bodies. Similar results were observed with αTAG72-osmY and αHer2-osmY. These results suggest that the N-terminus of osmY fused with scFv produces a high yield of soluble, functional, and secreted scFv, and the osmY-based bacterial secretion system may be used for the large-scale industrial production of low-cost αEGFR protein.

## Introduction

Single-chain variable fragments (scFvs) retain the original antigen-binding activity and possess several unique properties such as small size, easy engineering, good tumor penetration, rapid blood clearance, and low antigenicity [1]–[3]. Therefore, they have been widely used in industrial, medical diagnostic, and research and therapeutic applications [4]–[6]. Currently, there is a need to develop cost-effective approaches for the mass production of scFvs. When compared with other expression strategies, the bacterial expression system is the most economic strategy for the production of scFv antibodies [7], [8]. However, the mass production of scFvs in the bacterial cytoplasm or periplasmic space often leads to protein misfolding, aggregation, and accumulation within inclusion bodies [9], [10]. To circumvent these problems, Jurado *et al.* showed that if the culture temperature is reduced to 16°C, the ratio of the soluble fraction versus whole cell protein extracts of Trx-scFv B7 increased 6-fold, but Trx-scFv B7 in whole cell protein extracts also decreased by approximately 80% [11]. This indicates that lower growth temperature enhanced the solubility of scFv but reduced the total protein production. In addition, Hu *et al.* demonstrated that the proper folding of recombinant scFv was enhanced when domoic acid-binding scFv was co-expressed with the *Escherichia coli* chaperone DnaKJE. Although a 35% increase in the yield of the soluble fraction was achieved by this method, the production process in the bacteria was more complicated [12]. In contrast, protein purification from bacterial extracts has been associated with a high risk of contamination, posing additional challenges in acquiring highly pure proteins [13], [14]. The secretion of scFvs into the LB medium would enhance the proper folding of recombinant scFvs, prevent protein contamination, and simplify the protein purification process to potentially allow large-scale cost-effective production of scFvs.

**Figure 1:**
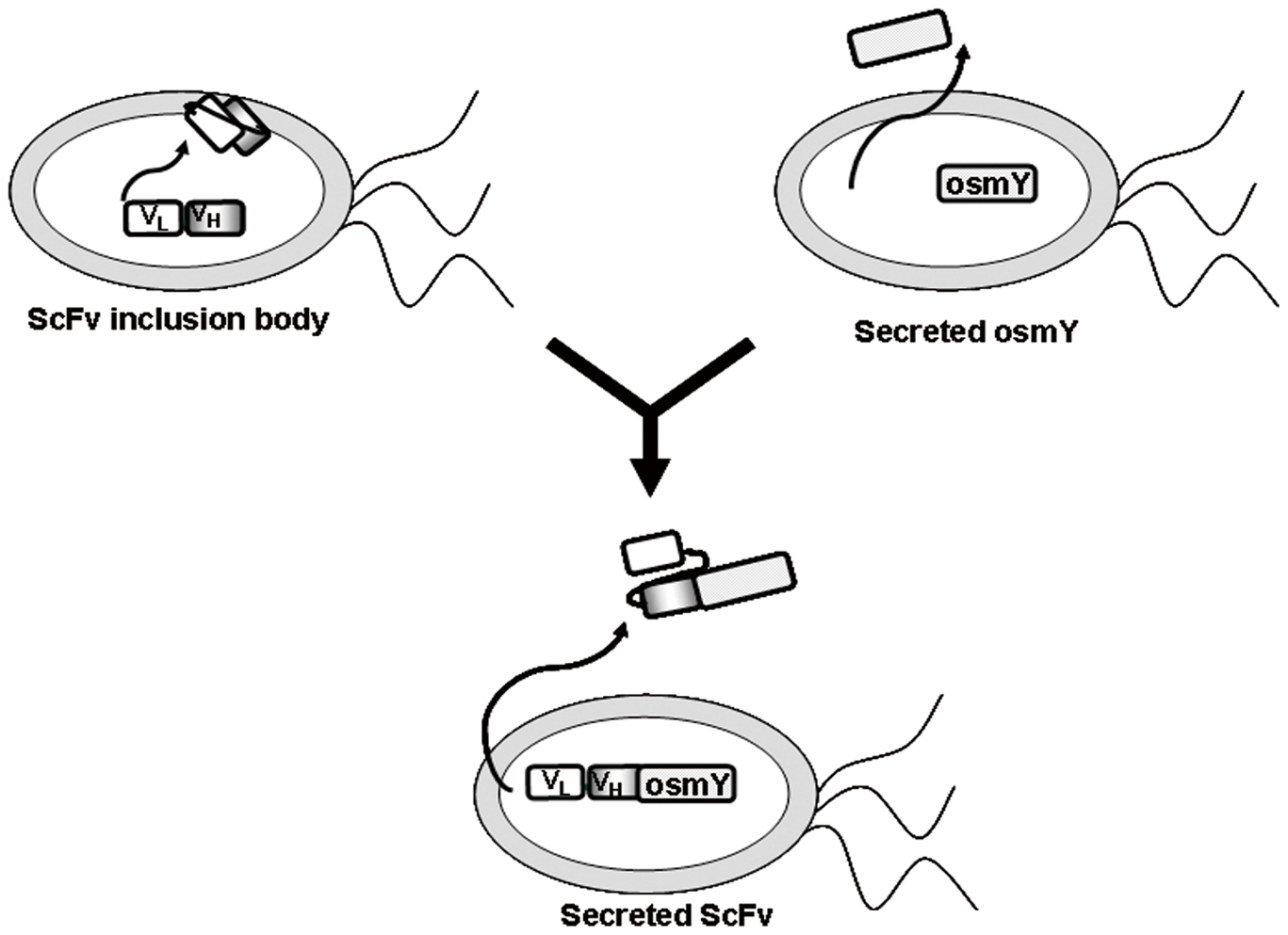
Development of a bacterial secretion system for the production of a soluble and secreted single-chain antibody. scFv was fused with the bacterial secretory carrier protein osmY to produce a good yield of soluble scFv secreted into the LB medium and to circumvent scFv inclusion body formation in the cytoplasm.

In this study, we developed a protein secretion system based on the fusion of scFvs with bacterial osmotically inducible protein Y (osmY), a bacterial secretion carrier, which produced a good yield of soluble scFv that was secreted into the LB medium (Fig. 1). The anti-EGFR scFv (αEGFR) and other scFvs (αTAG72, αHer2) were fused with the N- or C-termini of osmY or ***periplasmic leader sequence (PelB)*** to generate αEGFR-osmY, osmY-αEGFR, and conventional ***pelB-***α***EGFR***, respectively. To examine the expression of these αEGFR fusion proteins, the plasmids were transformed into *E. coli* BL-21 (DE3) to form αEGFR-osmY/BL21, osmY-αEGFR/BL21, and pelB-αEGFR/BL21, respectively. To determine the presence of αEGFR-osmY, osmY-αEGFR, and pelB-αEGFR, the growth medium, soluble lysate, and inclusion bodies were harvested for western blot analysis. Simultaneously, the function of the secreted αEGFR-osmY and osmY-αEGFR was examined by enzyme-linked immunosorbent assay (ELISA). Furthermore, the effect on antigen-binding activity was verified after the fusion of αEGFR with the N- or C-terminus of osmY. Both αEGFR-osmY and osmY-αEGFR were purified under non-denaturing conditions, whereas the control scFv pelB-αEGFR was purified under denaturing/refolding conditions. The functions of these scFvs were confirmed by ELISA. The approach adopted in the present study may provide a valuable system for the large-scale low-cost production of functional scFvs.

## Materials and Methods

### Bacteria and Cell Line

*E. coli* BL21 [F-ompT hsdSB (rB^−^, mB^−^) gal dcm (DE3), Novagen, San Diego, USA] was used in this study. MDA-MB-468 and SK-BR-3 human breast cancer cells (American Type Culture Collection, Manassas, VA, USA) were cultured in Dulbecco’s minimal essential medium (Sigma, St Louis, MO, USA) supplemented with 10% heat-inactivated bovine serum, 100 units/mL penicillin, and 100 µg/mL streptomycin (Gibco Laboratories, Grand Island, NY, USA) at 37°C in a humidified 5% CO_2_ atmosphere.

### Gene Construction of pET22b-osmY-αEGFR, pET22b-αEGFR-osmY, and pET22b-pelB-αEGFR

OsmY was amplified from *E. coli* BL21 genomic DNA by polymerase chain reaction (PCR), and the restriction sites *Nde*I, *Hind*III, *Sfi*I, and *EcoR*I were introduced using the following primers: osmY-*Sfi*I-*EcoR*I, 5′-CATATGACTATGACAAGAGTGAAGATTTCGAAAACTCTGCTGGCTGTAATGTTGACCTCTGCCGTCGCGACCGGCTCTGCCTACGCGGGCCCAGCCGGCCGAATTCGAAAACAACGCGCAG-3′ and 5′-AAGCTTGTGGTGGTGGTGGTGGTGCTTAGTTTTCAGATCA-3′; SfiI-*EcoR*I-osmY, 5′-CATATGACTATGACAAGACTG-3′ and 5′-AAGCTTGTGGTGGTGGTGGTGGTGGAATTCGGCCGGATGGGCCCTTAGTTTTCAGATCA-3′; and pelB-*Sfi*I-*EcoR*I, 5′-CATATGAAATACCTGCTGCCGACCGCTGCTGGTCTGCTGCTCCTCGCGCCCAGCCGGCGATGGCCATGGATATCGGA-3′ and 5′-AAGCTTGTG GTGGTGGTGGTGGTGGAATTCGGCCGGATGGGCCGAATTAATTCCGATATCC-3′. PCR fragments were digested with *Nde*I and *Hind*III, and then cloned into pET22b(+) (Novagen) to form pET22b-osmY-*Sfi*I-*EcoR*I, pET22b-*Sfi*I-*EcoR*I-osmY, and pET22b-pelB-*Sfi*I-*EcoR*I. The coding sequence of αEGFR was PCR amplified using the plasmid h528 (a generous gift from Dr. Makabe) [15] as a template, and the restriction sites *Sfi*I and *EcoR*I were introduced using the following primers: 5′-ACGCGTCGACGCGGCCCAGCCGGCCGATATTGTGATGACCCAGAGC-3′ and 5′-GGAATTCCGAGCTCACGGTAACCAG-3′. PCR fragments were digested with *Sfi*I and *EcoR*I and cloned into pET22b-osmY-*Sfi*I-*EcoR*I, pET22b-*Sfi*I-*EcoR*I-osmY, and pET22b-pelB-*Sfi*I-*EcoR*I to form pET22b-osmY-αEGFR, pET22b-αEGFR-osmY, and pET22b-pelB-αEGFR, respectively.

### Confirmation of pET22b-osmY-αEGFR, pET22b-αEGFR-osmY, and pET22b-pelB-αEGFR Gene Expression by Western Blot Analysis

The constructs pET22b-osmY-αEGFR, pET22b-αEGFR-osmY, and pET22b-pelB-αEGFR were transformed into *E. coli* BL21 to obtain pET22b-osmY-αEGFR/BL21, pET22b-αEGFR-osmY/BL21, and pET22b-pelB-αEGFR/BL21 cells, respectively. The scFv fusion protein expression was detected by western blot analysis using a mouse anti-histidine (His)-tag antibody (MCA1396, Serotec Raleigh, NC). The transformed BL21 cells were grown to an O.D._600nm_ of 0.7, and then protein expression was induced by adding 0.2 mM isopropyl-beta-D-thiogalactopyranoside (IPTG) to the cells at room temperature (RT) for 4 h. Subsequently, a 100-µL aliquot of the bacterial suspension was harvested and immediately mixed with 20 µL of 6× reducing sample buffer, and 20 µL of this mixture was loaded onto SDS-PAGE gel (3% stacking gel; 10% running gel). The proteins were transferred onto nitrocellulose membranes (Hybond C-extra, Amersham), and the membranes were blocked with phosphate buffered saline-0.05% Tween (PBST) containing 5% non-fat milk for 1 h at RT. The blocked membranes were then incubated with the mouse anti-His tag antibody in PBST containing 2.5% non-fat milk (1∶2,000 dilution) for 1 h. After washing, the membranes were incubated with horseradish-conjugated goat anti-mouse IgG (1∶2,000) in the same buffer for 1 h. After extensive washing in PBST, the membranes were developed using an ECL luminescence kit (Millipore, Bedford, MA, USA) and were exposed to X-ray film.

### Analysis of Solubility and Secretion of pET22b-osmY-αEGFR, pET22b-αEGFR-osmY, and pET22b-pelB-αEGFR in the Bacteria by Western Blot Analysis

The transformed bacterial pET22b-osmY-αEGFR/BL21, pET22b-αEGFR-osmY/BL21, and pET22b-pelB-αEGFR/BL21 cells were grown to an O.D._600nm_ of 0.7, and then protein expression was induced by adding 0.2 mM IPTG to the cells at RT for 4 h. To compare the protein quantity in the 3 different fractions, 20 mL of the LB culture broth of each group was first centrifuged at 6,000 rpm for 20 min at 4°C to separate the bacteria from the growth medium. The growth medium was then filtered through a 0.22-µm syringe filter to remove the bacterial cells that did not pellet out before concentration. The concentration conditions for each group were as follows: the media of pET22b-osmY-αEGFR/BL21, pET22b-pelB-αEGFR/BL21, and BL21 were concentrated 100-fold (from 20 to 0.2 mL), whereas that of pET22b-αEGFR-osmY/BL21 was concentrated 10-fold (from 20 to 2 mL). To analyze the solubility of these proteins, the bacterial pellet was sonicated 40 times at 10-s pulses in 20 mL of PBS. The cell lysates were centrifuged at 10,000 rpm for 20 min at 4°C to separate the supernatant (soluble protein) from the pellet (insoluble protein). Approximately 20 mL of PBS was added to the pellet, which was then resuspended by vortexing. Next, 100 µL of the concentrated growth medium, bacterial supernatant (soluble protein), and pellet (insoluble protein) were mixed with 20 µL of 6× reducing sample buffer, and a 20-µL aliquot was subjected to SDS-PAGE and western blot analysis. The distribution of pET22b-osmY-α EGFR, pET22b-αEGFR-osmY, and pET22b-pelB-αEGFR in the bacteria was observed and the intensity was estimated using a densitometer (Gel-Pro analyzer software from ***Media Cybernetics***).

### Analysis of the Function of Secreted osmY-αEGFR, αEGFR-osmY, and Refolded pelB-αEGFR by ELISA

MDA-MB-468 cells (10^5^ cells/well) were grown overnight in 96-well microtiter plates precoated with 10 µg/mL of poly-L-lysine (50 µL/well) for 30 min at 37°C. Then, glutaraldehyde (0.125%, 50 µL/well) was added to the plates and incubated at RT for 15 min. After washing once with PBS, 0.1 M glycine (100 µL/well) was added to the plates and incubated for 30 min at RT. Subsequently, the plates were washed 2 times with PBS and blocked with 200 µL/well dilution buffer (5% skim milk in PBS) at 4°C overnight. Then, 50 µL of the secreted osmY-αEGFR, αEGFR-osmY, and refolded pelB-αEGFR were added to the plates and incubated for 1 h at RT. The plates were washed 3 times with PBS and 50 µL/well of the mouse anti-His tag antibody (1∶2000 dilution) was added soaked in 2% skim milk, and incubated for 1 h at RT. After 3 washes with PBS, HRP-conjugated goat anti-mouse IgG Fc antibody in 50 µL of dilution buffer was added to the plates and incubated for 1 h at RT. The plates were washed as previously described and the bound peroxidase was measured by adding 150 µL/well of 2,2′-azinobis (3-ethylbenzthiazoline-6-sulfonic acid) (ABTS, Sigma-Aldrich) at a concentration of 0.4 mg/mL in the presence of 0.003% H_2_O_2_ and incubating for 30 min at RT. Color development was measured at a wavelength of 405 nm using a microplate reader.

## Results

### Construction and Expression of αEGFR-osmY, osmY-αEGFR, and pelB-αEGFR

**Figure 2:**
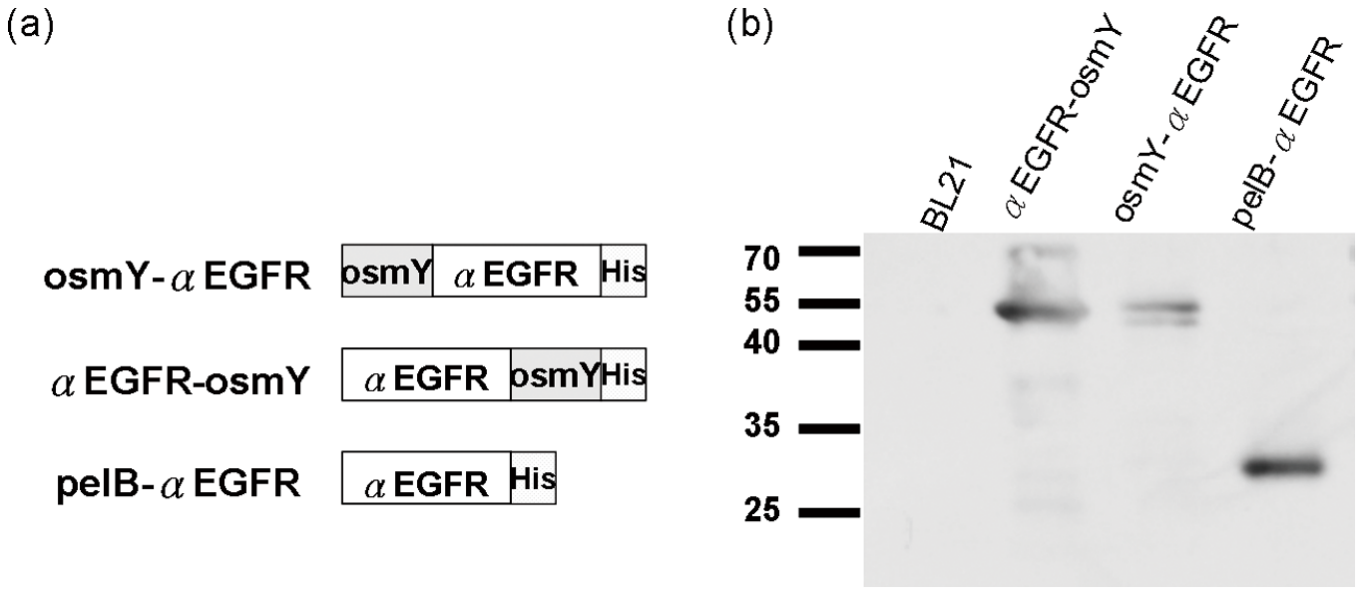
Construction and expression of secreted αEGFR. (a) αEGFR was fused with the N- or C-terminus of the *osmY* gene to form αEGFR-osmY and osmY-αEGFR fusion proteins, respectively. αEGFR expressed in the periplasmic space (pelB-αEGFR) was used as the control. H, Histidine tag. (b) αEGFR-osmY, osmY-αEGFR, and pelB-αEGFR plasmids were transformed into BL-21 to obtain αEGFR-osmY/BL21, osmY-αEGFR/BL21, and pelB-αEGFR/BL21 cells, respectively. The expression of αEGFR was confirmed by western blot analysis using an anti-histidine tag antibody. Lane 1, BL21 as negative control; Lane 2, αEGFR-osmY/BL21; Lane 3, osmY-αEGFR/BL21; and Lane 4, pelB-αEGFR/BL21.

To achieve secretion of scFvs into the LB medium, the gene encoding αEGFR was fused with the N- or C-terminus of the *osmY* gene to form the αEGFR-osmY and osmY-αEGFR fusion proteins (Fig. 2a). A plasmid for the expression of αEGFR in the periplasmic space (pelB-αEGFR) [16] was used as the control. To confirm the expression of different forms of αEGFR, these plasmids were transformed into the BL-21 (DE3) bacteria to obtain αEGFR-osmY/BL21, osmY-αEGFR/BL21, and pelB-αEGFR/BL21. The expression of these scFvs was detected by western blot analysis using an anti-His tag antibody, and αEGFR-osmY, osmY-αEGFR, and pelB-αEGFR were found to be expressed with the expected sizes of 51, 51, and 33 kDa, respectively (Fig. 2b).

### Solubility and Secretion of αEGFR-osmY, osmY-αEGFR, and pelB-αEGFR in the Bacteria

**Figure 3:**
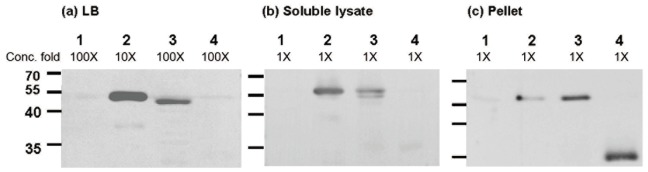
Solubility and secretion of αEGFR-osmY, osmY-αEGFR, and pelB-αEGFR in different components. The transformed cells were induced with IPTG and the presence of αEGFR-osmY, osmY-αEGFR, and pelB-αEGFR were detected by western blot analysis using an anti-histidine tag antibody in (a) concentrated LB medium, (b) soluble lysate, and (c) insoluble protein (pellet), as described in the Materials and Methods section. Lane 1, BL21 as negative control; Lane 2, αEGFR-osmY/BL21; Lane 3, osmY-αEGFR/BL21; and Lane 4, pelB-αEGFR/BL21.

To investigate whether the fusion of osmY could increase the protein solubility and secretion capacity of scFvs, western blot analysis using an anti-His tag antibody was conducted to detect scFv-osmY, osmY-scFv, and pelB-scFv in the concentrated LB medium, soluble lysate, or inclusion bodies of BL21. As shown in Fig. 3a, αEGFR-osmY and osmY-αEGFR, but not pelB-αEGFR, were present in the LB medium. The yield of αEGFR-osmY in the LB medium was 250-fold higher than that of osmY-αEGFR. Furthermore, αEGFR-osmY, osmY-αEGFR, and a small amount of pelB-αEGFR were present in the soluble lysate, and the yield of αEGFR-osmY in the soluble lysate was 20- and 250-fold higher than that of osmY-αEGFR and pelB-αEGFR, respectively (Fig. 3b). Most inclusion bodies contained pelB-αEGFR, and the abundance of pelB-αEGFR was approximately 10- and 4-fold higher than that of αEGFR-osmY and osmY-αEGFR, respectively (Fig. 3c). These results were similar to those observed for other scFvs (αTAG-72 and αHER2) (Table 1). The results indicate that the solubility and secretion capacity of scFvs were enhanced after fusion with osmY (N- or C-terminus), but not with pelB. Among the 2 fusion proteins, the one obtained by fusing N-terminus of osmY with scFv produced a higher yield of soluble and secreted scFvs.

**Table 1:** Subcellular localization of scFv fusion protein.

	pelB-scFv			osmY-scFv			scFv-osmY		
	LB (%)	S (%)	P (%)	LB (%)	S (%)	P (%)	LB (%)	S (%)	P (%)
αEGFR	0	20.00	80.00	16.70	50.00	33.30	33.30	50.00	16.70
αHer2	2.50	37.50	60.00	2.50	37.50	60.00	16.70	50.00	33.30
αTag-72	0	20.00	80.00	16.70	50.00	33.30	16.65	66.70	16.65

LB: growth medium. S: soluble lysate. P: pellet.

The distribution of osmY-scFv, scFv-osmY, and pelB-scFv in the bacteria was observed by western blot analysis, and the intensity was estimated by densitometry. The protein quantity was calculated on the basis of protein concentration folds and intensity ratios and presented as a percentage.

### Function of Secreted αEGFR-osmY, osmY-αEGFR, and pelB-αEGFR in the LB Medium

**Figure 4:**
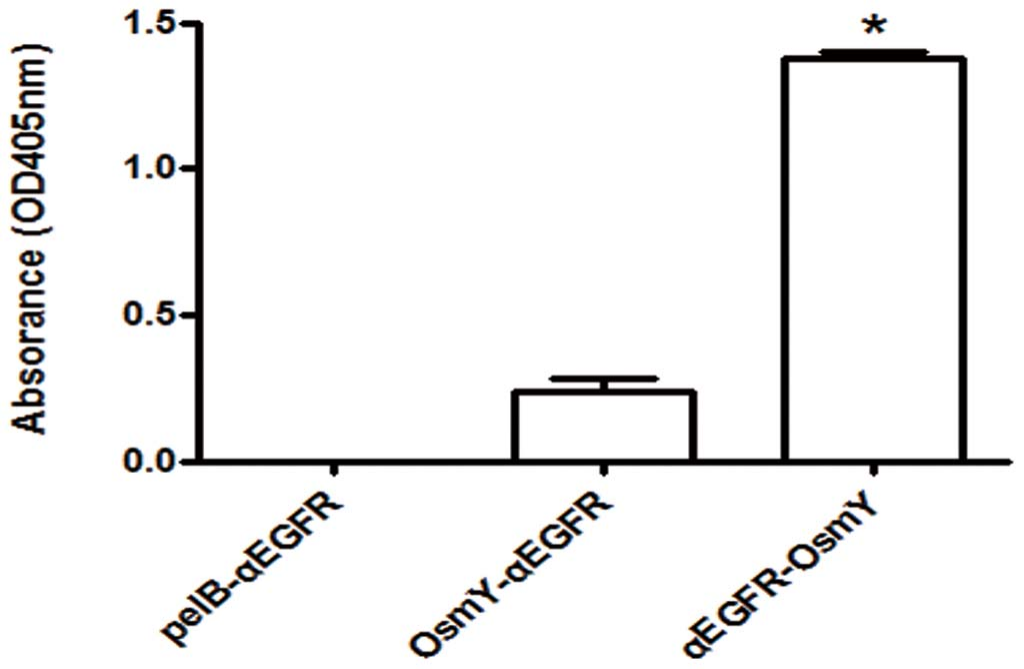
Function of secreted αEGFR-osmY, osmY-αEGFR, and pelB-αEGFR. The growth medium of αEGFR-osmY/BL21, osmY-αEGFR/BL21, and pelB-αEGFR/BL21 was added to the EGFR-positive MDA-MB-468 cells, and the binding activity of the αEGFR fusion protein was detected by ELISA using an anti-histidine tag antibody.

To verify whether the antigen-binding activity was retained in the secreted αEGFR-osmY, osmY-αEGFR, and pelB-αEGFR, the LB growth medium from these 3 groups was harvested. After incubation of the harvested LB medium with EGFR-positive MDA-MB-468 cells, the binding activity of αEGFR fusion proteins was detected by ELISA using an anti-His tag antibody. As shown in Fig. 4, the secreted αEGFR-osmY (1.38±0.02) and osmY-αEGFR (0.24±0.01), but not pelB-αEGFR (0.01±0.00), bound to the EGFR-positive MDA-MB-468 cells. These results indicate that the secreted αEGFR-osmY and osmY-αEGFR retained their ability to bind to EGFR.

### Comparison of the Antigen-binding Activity of αEGFR-osmY, osmY-αEGFR, and Refolded pelB-αEGFR

**Figure 5:**
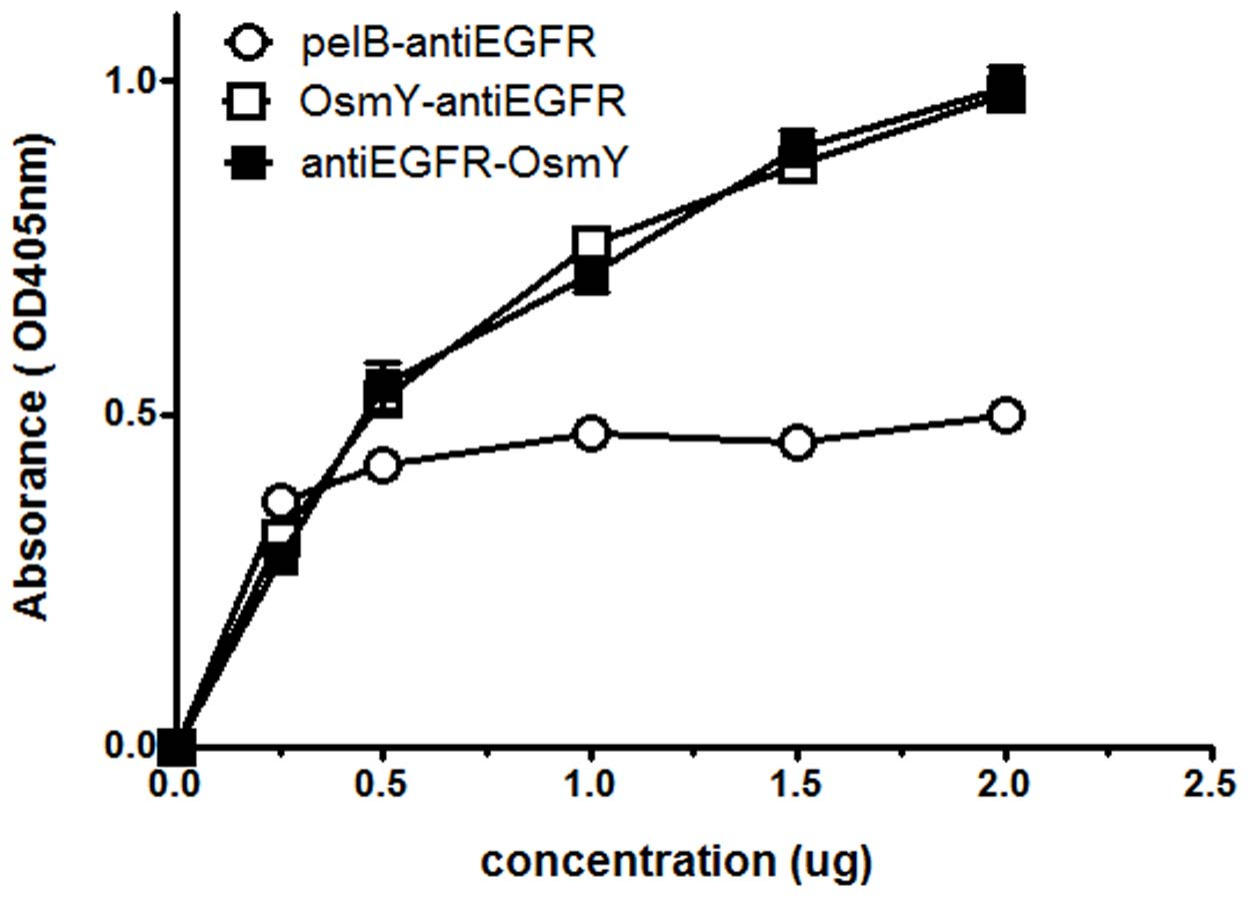
Antigen-binding activity of αEGFR-osmY, osmY-αEGFR, and refolded pelB-αEGFR. The fusion protein αEGFR-osmY and osmY-αEGFR were purified by using a Ni-column, and the control pelB-αEGFR was ***purified*** by using the Ni-column under denaturing/refolding conditions. The binding activities of different concentrations of αEGFR against EGFR-positive cells (MDA-MB-468) were determined by ELISA using an anti-histidine tag antibody.

To verify whether the antigen-binding activity of αEGFR was affected by fusion with the N- or C-terminus of osmY, αEGFR-osmY and osmY-αEGFR were purified using a Ni-column under non-denaturing conditions, whereas the control pelB-αEGFR was purified under denaturing/refolding conditions. The binding capacity of various concentrations of αEGFR scFvs to EGFR-positive cells (MDA-MB-468) was determined by ELISA using an anti-His tag antibody. Figure 5 shows that the slope of the binding curve for αEGFR-osmY (0.467±0.003) and osmY-αEGFR (0.456±0.008) was similar, but 2-fold higher than that for pel-αEGFR (0.174±0.008). Thus, fusion of αEGFR with either the N- or C-terminus of osmY did ***not affect*** its ***antigen-binding*** capacity. In addition, the binding activities of both the osmY-fused αEGFRs were better than that of conventional pelB-αEGFR purified under denaturing conditions.

## Discussion

In this study, we successfully developed an scFv secretion system on the basis of the fusion of scFvs with the bacterial secretory carrier protein osmY. Our results indicate that osmY increased the solubility of scFvs, thus circumventing the problems of misfolding, aggregation, and accumulation within inclusion bodies. The fusion of the N-terminus of osmY with scFvs produced the highest yield of soluble and secreted scFvs. In addition, the fusion with osmY (N- or C-terminus) did not affect the antigen-binding activity of αEGFR scFvs. Therefore, we propose that this bacterial secretory system is suitable for large-scale protein production.

Protein purification using an extracellular secretion system may circumvent several disadvantages when compared with the traditional cytoplasmic production system. First, without disrupting the bacterial outer membrane, the contamination of endotoxin and cytosolic proteins is significantly reduced [13], [14], [17]. Second, the use of *E. coli* K-12 significantly lowers the amount of endogenous proteins secreted into the growth medium under standard conditions [18]. Third, the risk of proteolysis in the cytosol is much lower when the bacteria secrete these proteins [13], . Taken together, protein purification from the growth medium is stable, simple, and easy to perform. In this study, we demonstrated that the fusion of scFvs with osmY greatly enhanced both the solubility and secretion efficiency as compared with the traditional methods of scFv construction (pelB-αEGFR).

The fusion of bacterial carrier proteins allows the secretion of various proteins into the growth medium. Zheng *et al*. identified the most efficient excreting fusion partner osmY from the extracellular proteome of the *E. coli* B strain BL21 (DE3). They demonstrated that several proteins fused with the C-terminus of osmY could be secreted into the growth medium, including *E. coli* alkaline phosphatase, *Bacillus subtilis* alpha-amylase, and human leptin [20]. In addition, Zheng *et al.* showed that xylanases could be secreted to the extracellular environment by fusing them with osmY [21]. Kotzsch *et al.* showed that various proteins fused with the C-terminus of osmY, enhancing the solubility and folding of proteins such as CXCL-9, NRN1, and ACVR1 [22]. However, the secretion of scFvs fused with osmY has yet not been investigated. In this study, we fused various scFvs to the N- or C-terminus of osmY to assess its function, solubility, and secretion. We observed an enhanced secretion of scFv-osmY fusion proteins as compared with scFvs fused with pelB. In addition, we observed that the fusion of αEGFR scFvs with the N-terminus of osmY greatly enhanced its solubility (>20-fold) and secretory efficiency (>250-fold) as compared with the fusion of αEGFR scFvs with the C-terminus of osmY. Similar results were also observed following comparisons of N- and C-terminal fusions with other osmY fusion proteins such as porcine circovirus type 2 (PCV2) capsid protein and cytolysin (cyt) (data not shown). Therefore, we conclude that the fusion of a target protein with the N-terminus of osmY offers the highest potential as a cost-effective strategy for the large-scale production of proteins.

The development of osmY-based bacterial secretion system has considerable potential for applications in industries. Some of the potential applications include the following. (1) Nervous necrosis virus (NNV) is a major viral pathogen that infects the larval stage of the grouper in aquaculture and causes serious economic loss [23], [24]. The use of an engineered probiotic that secretes a virus-neutralizing scFv may be able to prevent NNV outbreaks in aquaculture. (2) The PCV2 capsid protein has been produced from bacterial lysates and used as a vaccine against post-weaning multisystemic wasting syndrome [25], [26]. As the preparation of bacterial lysates is relatively complicated, the immune response against PCV2 is greatly diminished. However, secretion of PCV2 into a medium with lower endogenous proteins would enhance the quality and immunogenicity of PCV2. (3) Lignin peroxidases from fungi exhibit coal depolymerization activity, converting insoluble lignin into soluble polymers [27]. A probiotic could be modified to continuously release lignin peroxidase to depolymerize the lignin in the environment. (4) Yellow head virus (YHV) is an invertebrate nidovirus that caused high mortality in cultured black tiger shrimp (*Penaeus monodon*). Intorasoot *et al.* generated a refolded scFv antibody against YHV envelope glycoprotein 116 that detects YHV-infected shrimp 24 h post-infection and can potentially prevent YHV outbreaks [28]. If this scFv antibody is fused with osmY, we believe that the process of antibody purification will be simplified and the associated cost reduced. Therefore, the osmY-based bacterial secretion system may have great significance in industries. In contrast, this system may not be completely suitable for therapeutic proteins because osmY may elicit an immune response, thus requiring the removal of therapeutic proteins from osmY and subsequent purification. Such processes would increase the cost of producing the recombinant protein.

In summary, the osmY-based bacterial secretion system outlined in this study has the following important advantages: (1) it is not necessary to disrupt the bacterial outer membrane to generate a high protein yield; (2) there is a lower risk for cytosolic protein contamination and instead provides a simpler purification process; (3) protein loss from intracellular proteolysis is avoided; (4) the problem of limited periplasmic volume to continuously produce scFv fusion proteins is circumvented [29]; and (5) scFv fusion proteins in the growth medium are mature and are highly likely to undergo proper protein folding [14]. These results suggest that the N-terminus of osmY fused with scFvs would be useful for the large-scale production of functional, soluble, and secreted antibodies. This bacterial secretion system may have a potential to increase the mass production of proteins for a wide range of purposes, particularly for research and industrial applications.
